# Dietary Supplementation of Selenoneine-Containing Tuna Dark Muscle Extract Effectively Reduces Pathology of Experimental Colorectal Cancers in Mice

**DOI:** 10.3390/nu10101380

**Published:** 2018-09-27

**Authors:** Junko Masuda, Chiho Umemura, Miki Yokozawa, Ken Yamauchi, Takuya Seko, Michiaki Yamashita, Yumiko Yamashita

**Affiliations:** 1 Department of Medical Bioengineering, Graduate School of Natural Science and Technology, Okayama University, Okayama 700-0080, Japan; pmyu2alv@s.okayama-u.ac.jp; 2 Nutrition Act Co., Ltd., Tokyo 104-0061, Japan; m-yokozawa@n-act.co.jp (M.Y.); k-yamauchi@n-act.co.jp (K.Y.); 3 National Research Institute of Fisheries Science, Kanagawa 236-8648, Japan; sekotakuya@affrc.go.jp (T.S.); ymk0125@affrc.go.jp (Y.Y.); 4 Department of Food Science and Technology, National Fisheries University, Yamaguchi 759-6595, Japan; mic@fish-u.ac.jp

**Keywords:** colorectal cancer, selenoneine, selenoneine-containing tuna dark muscle extract, myeloid-derived suppressor cells, tumor growth, regulatory T cells

## Abstract

Selenoneine is an ergothioneine analog with greater antioxidant activity and is the major form of organic selenium in the blood, muscles, and other tissues of tuna. The aim of this study was to determine whether a selenoneine-rich diet exerts antioxidant activities that can prevent carcinogenesis in two types of colorectal cancer model in mice. We administrated selenoneine-containing tuna dark muscle extract (STDME) to mice for one week and used azoxymethane (AOM) and dextran sodium sulfate (DSS) for inducing colorectal carcinogenesis. Next, we examined the incidence of macroscopic polyps and performed functional analysis of immune cells from the spleen. In the AOM/DSS-induced colitis-associated cancer (CAC) model, the oral administration of STDME significantly decreased tumor incidence and inhibited the accumulation of myeloid-derived suppressor cells (MDSCs) while also inhibiting the downregulation of interferon-γ (IFN-γ) production during carcinogenesis. These results suggest that dietary STDME may be an effective agent for reducing colorectal tumor progression.

## 1. Introduction

Colorectal cancer (CRC) is the fourth-leading cause of the cancer-related death and the third-most diagnosed malignancy worldwide [1]. The tumor microenvironment consists of a variety of cancer-associated fibroblasts, endothelial cells, tumor-infiltrating lymphocytes, myeloid-derived suppressor cells (MDSCs), and macrophages, and the systemic immune response is altered by the secretion of cytokines and other mediators by tumor growth [2,3,4]. MDSCs represent a heterogeneous cell population whose members share a capacity to exert immunological suppressor functions [5,6], and whose numbers increase in the spleen with the growth of human and murine cancers [7,8]. In contrast, both CD4^+^ and CD8^+^ T cells, as well as CD4^+^ Foxp3^+^ regulatory T cells (Tregs) decrease significantly in the spleen, which leads to a reduction of all representative cytokines such as IFN-γ, IL-4, and IL-10 in allograft colorectal tumor models compared to normal controls [9].

Reactive oxygen species (ROS), which include hydrogen peroxide, hydroxyl radicals, superoxide anions, and peroxynitrites are chemically highly reactive, a property that is primarily because of their unpaired electrons (radicals) [10,11]. Excessive ROS production induces aberrant NF-κB activation that cause inflammatory bowel disease (IBD) or colitis-associated cancer (CAC) [12,13]. Some studies have shown that the intake of antioxidants inhibits IBD and CAC in mouse models [14,15,16]. In particular, the antioxidant ergothioneine scavenges intestinal oxidative stress after intracellular uptake by the organic carnitine transporter (OCTN1), which is expressed in the intestine [17].

Supplementation with inorganic and organic selenium compounds is considered anticarcinogenic at doses greater than those required to support the maximum expression of selenoproteins, which are generally regarded as completely responsible for the nutritional effects of selenium [18,19,20]. In the rat model, the addition of sodium selenite to the diet at the 2–5 ppm concentration was effective in preventing chemical carcinogenesis using 7,12-dimethylbenzanthracene [21]. Combs et al. have suggested that antioxidant selenium compounds present in foods are metabolized to yield selenide or methylated selenides [18,20].

An analog of ergothioneine, the selenium (Se)-containing antioxidant selenoneine (2-selenyl-Nα,Nα,Nα-trimethyl-l-histidine), is the major form of organic selenium in the blood, muscles, and other tissues of tuna [22]. The 50% radical scavenging concentration (RS_50_) of reduced selenoneine measured using with 1-diphenyl-2-picrylhydrazyl (DPPH) is 1.9 μM, while those of the water-soluble vitamin E-like antioxidant Trolox^TM^ and l-ergothioneine are 880 and 1700, respectively, indicating that selenoneine is a more effective antioxidant [22]. Selenoneine is taken up into human cells by OCTN1 where it efficiently reduces ROS levels and promotes cell proliferation [22,23], and it has been suggested that the intestinal absorption of ergothioneine by OCTN1 protects against the intestinal oxidative stress that induces colitis [17,24,25]. In addition, selenium can suppress carcinogenesis and tumor growth [26,27], suggesting that selenoneine may also be effective in this regard. However, the question of whether the dietary intake of selenoneine can inhibit carcinogenesis and tumorigenesis and influence systemic immunological responses is yet to be addressed.

In the present study, we utilized a diet of tuna dark muscle extract (STDME), which contains selenoneine, and determined whether its antioxidant properties are sufficient to suppress the development of experimental colon carcinogenesis in mice. The results of this study suggest that STDME inhibits the increase in MDSC activity that protects tumors from immunological attack.

## 2. Materials and Methods

### 2.1. Reagents, Antibodies (Abs), and Diet

Azoxymethane (AOM), retinoic acid (RA), 3-methylcholanthrene (MCA) and Giemsa stain were purchased from Sigma-Aldrich (St. Louis, MO, USA). Dextran sodium sulfate (DSS) was purchased from MP Biomedicals (Santa Ana, CA, USA). Anti-mouse CD3ε (145-2C11) and CD4 (RM4-5) were purchased from BD Biosciences (San Jose, CA, USA). Anti-mouse CD16/CD32 (93), and Gr-1 (RB6-8C5) mAbs were purchased from eBioscience (San Diego, CA, USA). Anti-CD8α (53–6.7), Foxp3 (3G3), and 7-amino-actinomycin D (7AAD) were from Tonbo Biosciences. The components of the AIN-93M standard diet were purchased from Oriental Yeast Co., Ltd. (Tokyo, Japan).

### 2.2. Preparation of STDME Diet

The STDME was prepared from the frozen dark muscle of bigeye-tuna. After thawing, the muscle was minced and boiled in three volumes of milli-Q^TM^ water for 5 min. The boiled aqueous extract was filtrated with celite (Showa Chemical Co., Tokyo) and Sepabeads SP-207 (Sigma-Aldrich Co., St. Louis, MO, USA) resins, and vacuum-concentrated to Brix55, stored at −80 °C until use. The selenoneine in STDME and feed were determined by LC-ICPMS described in previous work [28]. The final composition of the STDME and the experimental diets are shown in Table 1 and Table 2.

### 2.3. Animals

Animals were obtained from Charles River Inc. (Kanagawa, Japan). Wild-type (WT) BALB/c female mice (age, five weeks; weight, 17–19 g) were maintained under specific pathogen-free conditions at the Tsushima-kita Branch, Department of Animal Resources, Advanced Research Center, Okayama University. Animals were kept at 22 °C–26 °C and 50% humidity with a 12 h light/dark cycle and were fed a standardized diet with ad libitum access to autoclaved tap water. All animal experiments were reviewed and approved by the ethics committee (Animal Care and Use Committee) for animal experiments of Okayama University under the project identification codes IDs OKU-2015229, OKU-2017549 OKU-2018396 OKU-2018450 and OKU-2018526.

### 2.4. AOM/DSS-Induced CAC Model

**Figure 1:**
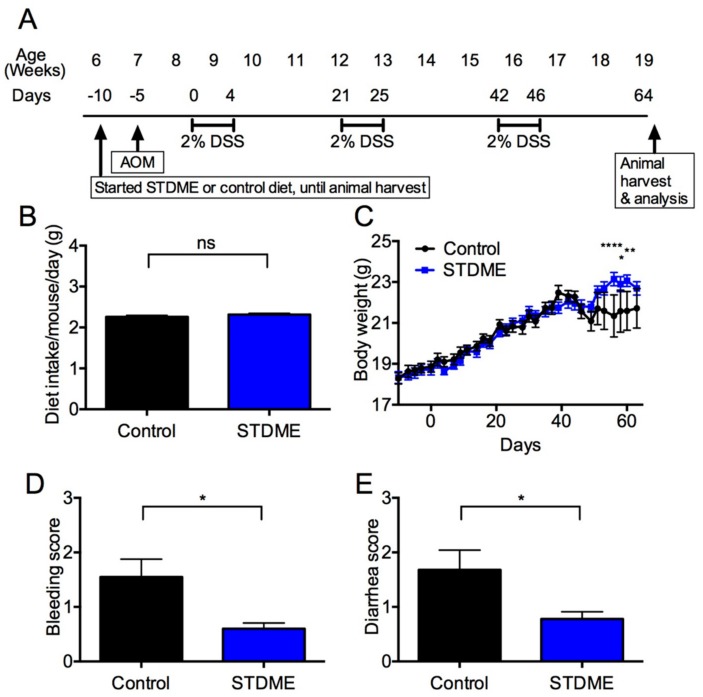
AOM/DSS-induced CAC in STDME-fed mice. (**A**) Schematic time schedule of change to STDME or AIN-93M (control) diets, intraperitoneal injection of the procarcinogen AOM, and DSS administration for this CAC model; (**B**–**E**) Dietary intake (**B**), body weight (**C**), bleeding score (**D**), and diarrhea score (**E**) in STDME-fed and control mice at 64 days after induction of CAC by AOM/DSS. Data are presented as mean ± SEM and were assessed using the Mann-Whitney *U*-test (**B**,**D**, and **E**) and two-way ANOVA (**C**). * *p* < 0.05; ** *p* < 0.01; **** *p* < 0.0001; ns = not significant; *n* = 10/group; AOM, azoxymethane; CAC, colitis-associated cancer; DSS, dextran sodium sulfate; STDME, selenoneine-containing tuna dark muscle extract.

Six-week-old BALB/c mice were randomized to a STDME in AIN-93M diet or a control diet (AIN-93M alone) with free access to food and water during experiments. The induction of colitis-associated carcinogenesis was performed as previously described [13]. Briefly, the mice were intraperitoneally injected with AOM (10 mg/kg) dissolved in PBS. Five days later, 2% (*w*/*v*) DSS (MW 36,000–50,000; ICN Biomedicals, Aurora, OH, USA) in drinking water was given for five days followed by 16 days of regular water. This cycle was repeated three times. Body weight and dietary intake were monitored every 2–3 days, and mice were sacrificed at 64 days for the analysis (Figure 1A). Bleeding and diarrhea scores were determined as previously described [29].

### 2.5. Splenocyte Cultures and Assays for Cytokine Levels

Splenocytes were isolated and cultured as previously described [9,30]. Briefly, splenocytes (4 × 105 cells/well) were cultured for 48 h on flat-bottomed 96 well plates (Corning Costar, Cambridge, MA, USA) coated with 5 μg/mL anti-CD3ε monoclonal antibody (mAb) in 200 μL Roswell Park Memorial Institute (RPMI) 1640 medium (Sigma-Aldrich, St. Louis, MO, USA) containing 50 μM 2-mercaptoethanol (Nacalai Tesque, Inc., Kyoto, Japan) and supplemented with 10% (*v*/*v*) heat-inactivated fetal bovine serum (FBS) (SAFC Biosciences, Lenexa, KS, USA) and 1% (*v*/*v*) antibiotic-antimycotic solution (10,000 U/mL penicillin, 10,000 μg/mL streptomycin and 25 μg/mL amphotericin B; Life Technologies, Gaithersburg, MD, USA). The cultures were incubated in a humidified atmosphere of 5% CO_2_ at 37 °C. After 48 h, IFN-γ, IL-4, and IL-10 levels in culture supernatants were evaluated using cytokine-specific enzyme-linked immunosorbent assays (ELISAs) that are commercially available from BD Biosciences.

### 2.6. Flow Cytometry (FCM)

Splenocytes (1 × 10^6^) were incubated with anti-CD16/CD32 mAb for 20 min on ice. Then, MDSCs were stained with anti-Gr-1 and CD11b mAbs. T cells were stained using anti-CD4, CD25, and CD8 mAbs for 30 min on ice, fixed with FACS Lysing Solution (BD Biosciences) for 10 min at room temperature (RT), permeabilized with FACS Permeabilizing Solution 2 (BD Biosciences) for 10 min at RT, and then stained with anti-Foxp3 mAb for 30 min. The stained cells were analyzed using an Accuri™ flow cytometer (BD Biosciences) and FlowJo Software (Treestar, Inc., San Carlos, CA, USA).

### 2.7. Cell Culture

A v-Ha-ras-transfected BALB/c 3T3 cell line, Bhas 42 (JCRB No. 0149), was provided by the Japanese Cancer Research Resources Bank (JCRB) and maintained in Dulbecco’s modified Eagle’s medium/Ham’s F12 (DMEM/F12) medium supplemented with 5% (*v*/*v*) FBS (Biowest, Nuaillé, France) and 1% (*v*/*v*) antimycotic antibiotic-solution (Nacalai Tesque, Inc.). The cultures were maintained in a humidified atmosphere of 5% CO_2_ at 37 °C.

### 2.8. Crystal Violet Assay

Bhas 42 cells (2 × 10^3^ /mL) in 100 μL culture medium were cultured for four days on 96 well plate prior to selenoneine treatments. The cells were then cultured with a serial concentration of selenoneine for 7 days. The plates were aspirated, washed with PBS, fixed by methanol, and stained with 0.1% crystal violet for 30 min. OD readings were taken at 540 nm. The relative cell growth rate (%) was obtained by the following equation:

(1) $$\%\;\mathrm{of}\;\mathrm{relative}\;\mathrm{cell}\;\mathrm{growth}=\frac{\mathrm{ODtreated}-\mathrm{ODblank}}{\mathrm{ODmedium}-\mathrm{ODblank}}\times 100$$

### 2.9. Cell Transformation Assay (CTA)

Bhas 42 cells (4 × 10^3^) in 2 mL culture medium were cultured for 24 h on six well plate prior to selenoneine or RA treatments. For growth phase, the cells were treated with 1 μg/mL MCA for three days, then treated 1 μM selenoneine or 0.3 μM RA for 10 days (stationary phase). The cells were replaced to flesh media with selenonein or RA every three days during this period, and then maintained in only culture medium for next 7 days. The cells were fixed by methanol, then stained with 5% (*v*/*v*) Giemsa to visualize and count transformed foci of cells as previously described [31].

### 2.10. Statistical Analyses

Statistical analyses were performed using the Student’s two-tailed *t*-tests, Mann-Whitney *U*-test and two-way ANOVA. All analyses were performed using GraphPad Prism Software Version 6 (GraphPad Software Inc., San Diego, CA, USA). *p*-values < 0.05 were considered to be statistically significant.

## 3. Results

### 3.1. Composition of the STDME Diet

As shown in Table 1, harmful heavy metals such as mercury and cadmium were largely eliminated during the preparation process of STDME from tuna dark muscle. The STDME diet contained 4.71% (*w*/*w*) STDME and 0.28 mg/100 g selenoneine (Table 2). The calories of STDME diet was adjusted by the amount of cornstarch to the same as the control diet.

### 3.2. Daily Intake of STDME Causes No Observable Toxicity or Disorders

The CAC mouse model was induced by an injection of procarcinogen AOM following the administration of DSS drinking water to elicit colitis for three cycles. Since differences in dietary intake amount can cause carcinogenesis, we measured the daily intake of STDME from six weeks to 19 weeks during the development of CAC. The STDME dietary intake per mouse per day was 2.26 ± 0.04 g during experimental carcinogenesis induction, and there was no difference compared with the control group (2.32 ± 0.03 g, Figure 1B). These results suggest that STDME administration does not cause observable toxicity or disorders during AOM/DSS-induced colon carcinogenesis.

### 3.3. Daily Intake of STDME Protects against Weight Loss in Experimental AOM/DSS-Induced Colon Carcinogenesis

**Figure 2:**
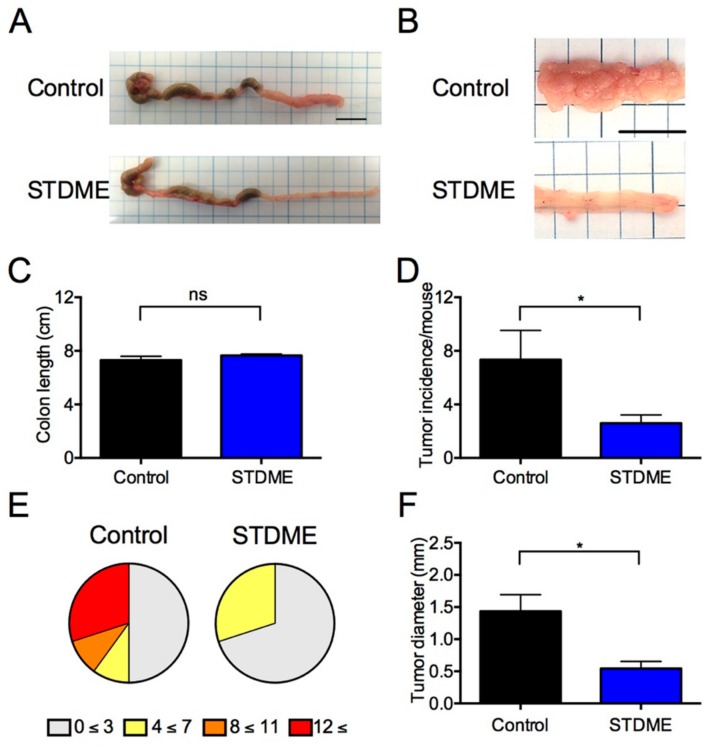
An STDME diet protects against AOM/DSS-induced CAC. (**A**–**D**,**F**) Representative pictures of colon (**A**), macroscopic polyps in the distal and mid-colons (**B**) and quantification of colon length (**C**) from STDME-fed mice compared with control mice; (**D**–**F**) The number of macroscopic polyps (**D**,**E**), and tumor diameter (**F**) were quantified. Data are presented as mean ± SEM and were assessed by a Mann-Whitney *U*-test. * p < 0.05; ns = not significant; *n* = 10/group. Each scale bar indicates 10 mm. STDME, selenoneine-containing tuna dark muscle extract.

Although the control group exhibited significant body weight loss, bleeding, and diarrhea after the third round of DSS treatment, the STDME group had significantly regained body weight, bleeding and diarrhea (Figure 1C–E). At the end of the experiment, the mice were sacrificed and tumors were found to be confined to the distal colon (Figure 2A,B). Despite colon length being the same (Figure 2C), tumor incidence (Figure 2D,E) and maximal cross-sectional diameter (Figure 2F) of macroscopic polyps were significantly lesser in STDME-fed mice. Taken together, these results suggest that the oral administration of STDME protects mice from AOM/DSS-induced CAC.

### 3.4. Daily Intake of STDME Suppresses Splenic MDSC Accumulation in Experimental AOM/DSS-Induced Colon Carcinogenesis

**Figure 3:**
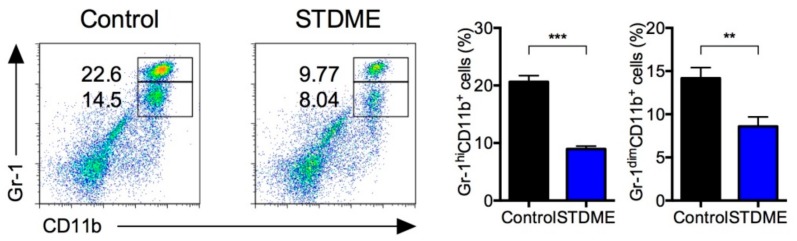
Suppression effect of splenic MDSCs in STDME-fed mice after AOM/DSS-induced CAC. Splenocytes from control or STDME-fed CAC-induced mice were isolated. The expression levels of Gr-1 on CD11b^+^ cells were analyzed by flow cytometry. Each bar represents mean ± SEM and data were assessed by a Mann-Whitney *U*-test. ** *p* < 0.01; *** *p* < 0.001; ns = not significant; *n* = 10/group. STDME, selenoneine-containing tuna dark muscle extract.

Given the protective effect of STDME against CAC, we next examined the accumulation of MDSCs in the spleen. MDSCs were identified as two populations by the co-expression of the myeloid lineage differentiation antigens Gr-1 and CD11b [9,30,32]. The percentages of Gr-1^dim^ CD11b^+^ and Gr-1^hi^ CD11b^+^ MDSCs in STDME-fed mice were significantly lower than those in the control group (Figure 3). These results indicate that the oral administration of STDME inhibits the accumulation of splenic MDSCs that is associated with regulation of T cell function by AOM/DSS-induced CAC.

### 3.5. Daily Intake of STDME Inhibits the Decrease in Splenic Regulatory T Cells in Experimental AOM/DSS-Induced Colon Carcinogenesis

**Figure 4:**
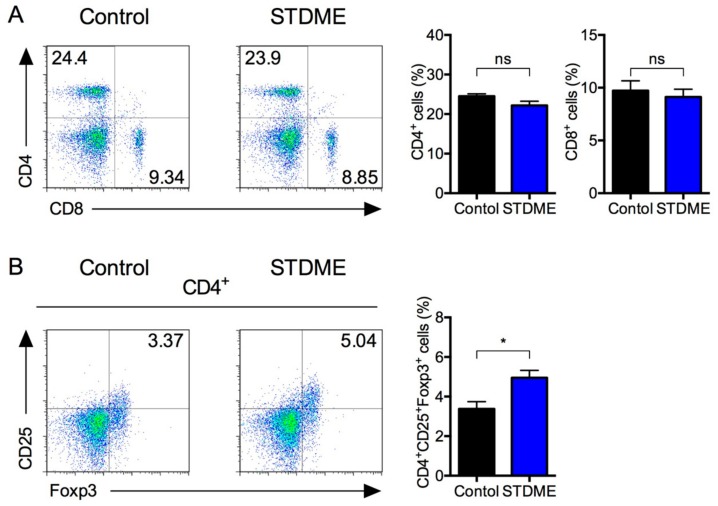
Splenic T cell subsets in STDME-fed mice after induction of AOM/DSS-induced CAC. Splenocytes from control or STDME-fed CAC-induced mice were isolated. Representative dot plot of CD4^+^, CD8^+^ and CD4^+^CD25^+^Foxp3^+^ splenocytes analyzed by flow cytometry. Each bar represents the mean ± SEM and the data were assessed by a Mann-Whitney *U*-test. * *p* < 0.05; ns = not significant; *n* = 10/group. STDME, selenoneine-containing tuna dark muscle extract.

Next, we turned our attention to levels (percentages) of T cells and T cell subsets in the spleens of the CAC model mice. The percentage of CD4^+^ and CD8^+^ T cells in STDME-fed mice were unchanged compared with controls (Figure 4A). Interestingly, however, the percentage of CD4^+^ CD25^+^ Foxp3^+^ Tregs in STDME-fed mice was higher than that of the control (Figure 4B), suggesting that the oral administration of STDME suppresses the attenuation of T cell function associated with CAC.

### 3.6. Dietary Intake of STDME Enhances Systemic Antitumor Immunity in Experimental AOM/DSS-Induced Colon Carcinogenesis

**Figure 5:**
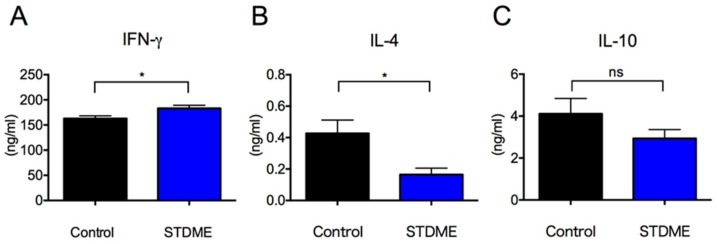
Cytokine production by splenocytes in STDME-fed mice after AOM/DSS-induced CAC. Splenocytes from control or STDME-fed CAC-induced mice were isolated and stimulated with plate-bound anti-CD3ε mAb for 48 h. (**A**–**C**) IFN-γ (**A**); IL-4 (**B**) and IL-10 (**C**) cytokines in culture supernatant were detected by ELISA. All data are presented as mean ± SEM and assed with a two-tailed Student’s *t*-test. * *p* < 0.05; ns = not significant; *n* = 10/group. STDME, selenoneine-containing tuna dark muscle extract.

Given that the differential regulatory cell responses in STDME-fed mice compared with controls, we next studied the cytokine responses of splenocytes in these mice. IFN-γ production was significantly higher in STDME-fed mice than in controls (Figure 5A). In contrast, IL-4 production in STDME-fed mice was significantly lower (Figure 5B). In addition, there was no statistically significant difference in IL-10 production (Figure 5C). Collectively, these results suggest that the oral administration of STDME enhances antitumor immunity by T cells in AOM/DSS-induced CAC.

### 3.7. Selenoneine Inhibits In Vitro Carcinogenesis

**Figure 6:**
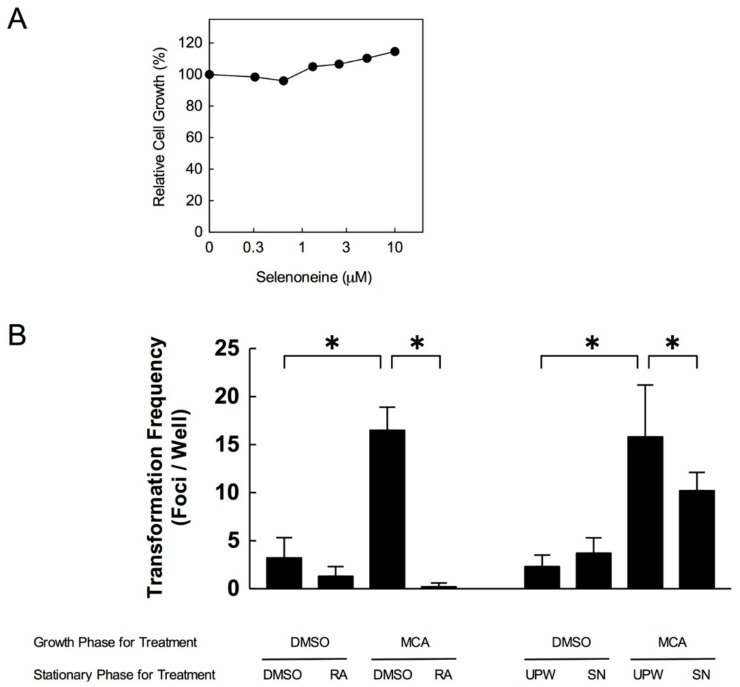
Relative cell growth and transformation frequency in Bhas 42 cells treatment with selenoneine. (**A**) The cells were cultured for 4 days, following at final concentration range of 0–10 μM for 7 days. The cell growth was determined by crystal violet assay; (**B**) The cells were cultured for 24 h. For growth phase, 1 μg/mL MCA was treated for 3 days, followed treatment with 0.3 μM RA in 0.5% (*v*/*v*) DMSO or 1 uM selenoneine in 1% (*v*/*v*) UPW for 10 days as stationary phase. After removal of RA of selenoneine from media, the cells were cultured for next seven days to observe the promotion activity in cell transformation. Each experiment was performed three times. All data are presented as mean ± SD and assed with a two-tailed Student’s *t*-test. * *p* < 0.05; DMSO, dimethyl sulfoxide; MCA, 3-Methylcholanthrene; RA, retinoic acid; SN, selenoneine; UPW, ultra pure water.

The CTAs using Bhas 42 cells are in vitro sensitive mutation tests to measure morphological transformation of the cells that are widely used to evaluate carcinogenicity [33]. In order to determine the proper concentration of selenoneine for CTAs, Bhas 42 cells were treated with purified selenoneine at the final concentration range of 0–10 μM for two days. Since the cell toxicity was not observed up to 10 μM (Figure 6A), we decided to treat 1 uM selenoneine for CTAs in the culture media.

To next examine carcinogenesis suppression activity of selenoneine, we performed CTAs. Since RA is an antioxidant that previously reported to inhibit the number of transformed frequency [34], we treated RA as positive control. As shown in Figure 6B, transformation frequency was promoted by MCA treatment and significantly attenuated by RA treatment. Consistently, similar results were also obtained by selenoneine treatment. These results suggest that selenoneine, itself, behaves as an antioxidant to inhibit carcinogenesis.

## 4. Discussion

Selenoneine is a novel organic selenium source found in the blood and muscle tissue of tuna. It exhibits a powerful radical scavenging ability and it is believed that dietary selenoneine is taken up by intestinal OCTN1, a common transporter of ergothioneine. However, the in vivo effects of a selenoneine diet have not been studied. Here, we demonstrated an inhibitory effect of an STDME diet in carcinogenesis, tumor formation, and tumor growth through the use of a murine colorectal cancer model.

As to the effect of STDME on these cancer models, it is possible that the not only antioxidative and other specific function in selenoneine but some biological effects caused by non-selenoneine chemical compositions contained in STDME might be involved in inhibitory effect. Although some groups reported that high protein intake induces cancer progression with destruction of anti-cancer immunesurveillance [33,35], STDME diet has only less than 1% difference in calories from that of control diet. While activated T cells must aerobically ferment glucose into lactate, low glucose induced metabolic stress that causes exhaustion of T cells in tumor. Activated T cells also require high proteins in an accordance with increase of metabolism [36]. However, how low-protein diet protect against cancer progression or anti-cancer immunesurveillance, especially in colon cancer is still unknown. STDME-diet is neither considered a protein-rich diet, nor induce the change in dietary ratio of carbohydrate, protein and fat. We show here inhibitory effect of splenic MDSC accumulation by STDME diet. The effects of STDME on this cancer model may be due not only to its antioxidative properties, but to other biological effects induced by its molecular structure. One of these effects was the inhibition of splenic MDSC accumulation by the STDME diet. Since MDSCs are myeloid precursors that expand during chronic inflammation and tumor growth, the accumulation of splenic MDSCs is observed in cancer model (Figure 3). MDSCs inhibit both adaptive and innate immunity through a variety of diverse mechanisms [9,30,37]. MDSCs that accumulate during tumor growth increase ROS production [38] and increased ROS suppresses antigen-specific activation of T cells [37,39] and differentiation of MDSCs into mature myeloid cells such as tumoricidal M1-like macrophages [37,40]. Moreover, the accumulation of ROS in MDSCs leads to an increased susceptibility to cancer metastasis [38,41]. Since the accumulation of ROS levels in MDSC is regulated by selenoenzymes such as glutathione peroxidase (GPx) and thioredoxin reductases (TrxR) [41], selenoneine might provide the selenium necessary to produce the selenocysteine that is the catalytic residue of these enzymes and so help to effectively scavenge ROS and prevent MDSC expansion.

Since inflammation occupies a key position in the development of sporadic CRC, primarily through the production of mutagenic ROS and reactive nitrogen species (RNS) [24], the CAC models we utilized in this study were an appropriate way of determining the effect of the antioxidant activity of selenoneine that causes the regulation of inflammation. Ergothioneine is an analog of selenoneine contained in food and is readily distributed throughout the small intestine after oral administration [24]. Intriguingly, recent reports suggest that OCTN1 functionality is expressed in activated lamina propria macrophages to increase ergothioneine absorption, which contributes to the suppression of the inflammation caused by DSS-induced colitis [42]. Therefore, selenoneine might have a similar inhibitory effect on the ROS-induced inflammation that causes CAC, resulting in the up-regulation of systemic immunity we demonstrated in the present study. Thus, after entering the systemic circulation via intestinal absorption, selenoneine might be incorporated into not only into red blood cells (RBC) [28], but also myeloid cells such as MDSCs. Further comprehensive evidences, such as expression of selenoproteine genes, GPx activitis, and peroxide contents in RBCs or myeloid cells monitoring with the serum levels of selenoneine by STDME diet or intake of purified selenoneine should be needed to fully understand the potential of selenoneine as a candidate diet for cancer prevention.

Tumorigenesis reduces the number of splenic T cells, including Tregs, which causes a decrease in antitumor cytokine production [9]. The population of Tregs increase by colitis along with increase of IFN-γ expressing effector T cells [43], but decreased by tumor growth [9]. Our present study indicated that STDME suppressed this attenuation of the antitumor activity of T cells in the spleen, while the percentage of CD4^+^ and CD8^+^ T cells was not affected. The intracellular ROS levels of T cells are strictly regulated through a variety of antioxidant systems. Although high amounts of extracellular ROS disable T cells, the amount of mitochondrial ROS production required to enhance T cell antitumor activity is not so high as to have a direct influence on tumor cells or other surrounding cells [44,45,46]. Excessive ROS produced by cancer cells can reach T cells and cause oxidative stress which might induce T cell hyporesponsiveness in cancer patients [47]. Since CD4^+^ and CD8^+^ T cells do not express OCTN1 [48], it is possible that an STDME diet preferentially eliminates ROS production from MDSCs and cancer cells without directly affecting T cell-dependent antitumor activity.

Numerous reports have shown that ROS production by cancer cells has both tumor-promoting and tumor-inhibiting properties [49]. Thus, the effect of dietary antioxidants on cancer remains controversial. For instance, some groups have shown that antioxidant dietary supplements help to reduce cancer risk [50,51,52], while other groups have concluded that they have both harmful and beneficial effects [53]. Especially in selenium supplementation, we must take into account for the reports that revealed no beneficial effect on population-based prostate cancer in the selenium and vitamin E cancer prevention trial (SELECT) and longer follow-up study [54,55], while other group reported that selenium leads apoptosis of cancer stem like cells in leukemia [56]. Since selenoneine is more powerful antioxidant than other selenium supplementation than ever used, STDME diet might show more valuable effect on cancer prevention. Thus, synergy effect of STDME with anti-cancer drugs on cancer therapy should be done for further study. Although low expression of OCTN1 is reportedly a strong predictor of poor event-free survival [57], the contribution of an STDME diet to cancer progression and whether MDSCs preferentially incorporate selenoneine compared with cancer cells are both still unknown. Further studies are required for determining if this is due simply to a difference in OCTN1 expression levels between MDSCs and tumor cells, or whether other alternative mechanisms exist.

## 5. Conclusions

In conclusion, we showed that an STDME diet protects against colorectal cancer. The underlying mechanism may involve the suppression of the splenic MDSC accumulation that promotes cancer progression by attenuation of antitumor activity. Moreover, selenoneine also affected the cells directly to inhibit in vitro carcinogenesis. Since selenoneine is highly concentrated in cells via OCTN1 activity [28], STDME might be effective as a dietary antioxidant. We anticipate that STDME will emerge as a functional food with excellent antioxidant properties, while further research to clarify the physiological roles of selenoneine to prevent carcinogenesis or tumor growth should be still required.

## Figures and Tables

**Table 1:** Approximate composition and energy content of STDME.

Components	Content (/100 g of STDME)
Energy (kcal)	361
Protein (g)	88.9
Fat (g)	0.60
Carbohydrate (g)	0.00
Ash (g)	18.1
Selenium (mg)	9.53
Selenoneine (mg)	11.4
Mercury (mg)	0.02
Heavy metals (Pb, Cu, Cd, Bi, and Sn)	Not detected

**Table 2:** Composition of experimental diets.

Ingredient	Control Diet (AIN-93M)	STDME Diet
Casein (%)	14	14
L-cystine (%)	0.18	0.18
Corn starch (%)	46.56	41.85
Pregelatinized corn starch (%)	15.5	15.5
Sucrose (%)	10	10
Soybean oil (%)	4	4
Cellulose (%)	5	5
Mineral mix (%)	3.5	3.5
Vitamin mix (%)	1	1
Choline bitartrate (%)	0.25	0.25
*Tert*-butylhydroquinone (%)	0.0008	0.0008
STDME (%)	0.00	4.71
Selenoneine (mg/100 g)	0.00	0.28
Selenium (mg/100 g)	0.01	0.45
Energy (kcal/100 g)	362	361
